# Rare variation: the absence of both the celiac trunk artery and the common hepatic artery

**DOI:** 10.1186/s12957-022-02858-x

**Published:** 2022-12-07

**Authors:** Zhenxing Zhang, Shan Wang, Minfeng Ye, Feng Tao, Danling Guo

**Affiliations:** 1 Department of Gastrointestinal Surgery, Shaoxing People’s Hospital Shaoxing Hospital, Zhejiang University School of Medicine, Shaoxing, 312000 China; 2 Department of Special Examination, Shaoxing People’s Hospital, Shaoxing Hospital, Zhejiang University School of Medicine, Shaoxing, 312000 China; 3 Department of Radiology, Shaoxing People’s Hospital, Shaoxing Hospital, Zhejiang University School of Medicine, Shaoxing, 312000 China

**Keywords:** Celiac trunk artery, Common hepatic artery, Anatomic variation, Anomaly, Laparoscopic radical resection of gastric cancer

## Abstract

**Background:**

Knowledge of celiac trunk anatomy is important in gastrointestinal surgery, hepatopancreatobiliary surgery, transplantation and interventional radiology. Variations in the celiac trunk are common and should be predicted prior to these interventions.

**Methods:**

A 58-year-old woman was admitted to our department for surgical treatment of gastric cancer (GC) confirmed by gastroduodenoscopy and gastric antrum biopsy. In the contrast-enhanced computed tomography (CT), we found an absence of both the celiac trunk artery (CA) and the common hepatic artery (CHA). Therefore, we used computerized three-dimensional (3D) vascular reconstruction technology to reconstruct the abdominal trunk and its branch vessels before operation.

**Results:**

Computerized 3D vascular reconstruction confirmed an extremely rare vascular anomaly: the absence of both CA and CHA. The splenic artery (SA) and gastroduodenal artery (GDA) originated from the abdominal aorta (AA). The left gastric artery (LGA) originated from the AA directly above the junction of SA and the GDA. The left hepatic artery (LHA) originated from the left gastric artery (LGA). The right hepatic artery (RHA) originated from the superior mesenteric artery (SMA). Laparoscopic radical resection of GC was performed. This anomaly was also confirmed intraoperatively. This patient was discharged on the 10th day after surgery without any postoperative complication. There were no signs of tumor recurrence during the 6-month follow-up.

**Conclusion:**

Correct identification of abnormal abdominal large blood vessels and their relationship with tumors before surgery is of great significance to avoid intraoperative blood vessel damage, major postoperative complications and the missing of lymph node dissection.

## Case report

**Fig. 1:**
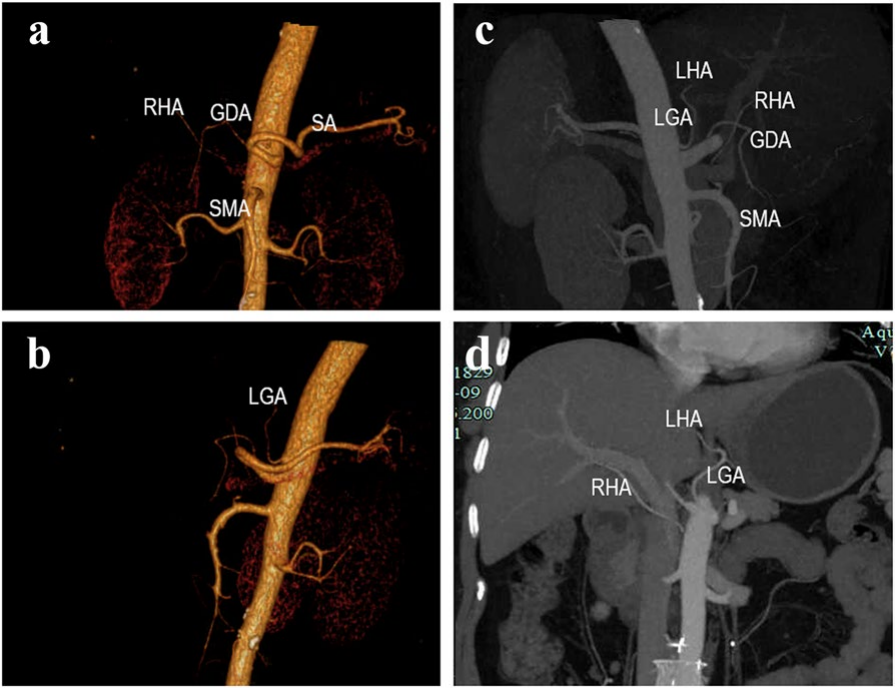
Computed tomography angiography (**c**, **d**) with reconstruction (**a**, **b**). An absence of the celiac trunk artery (CA) and the common hepatic (CHA) artery. The splenic (SA) and gastroduodenal (GDA) originates from the AA. The left gastric (LGA) originates from the abdominal aorta directly above the junction of the SA and GDA. At the same time, the left hepatic artery (LHA) originates from the LGA. The right hepatic artery (RHA) originates from the superior mesenteric artery

**Fig. 2:**
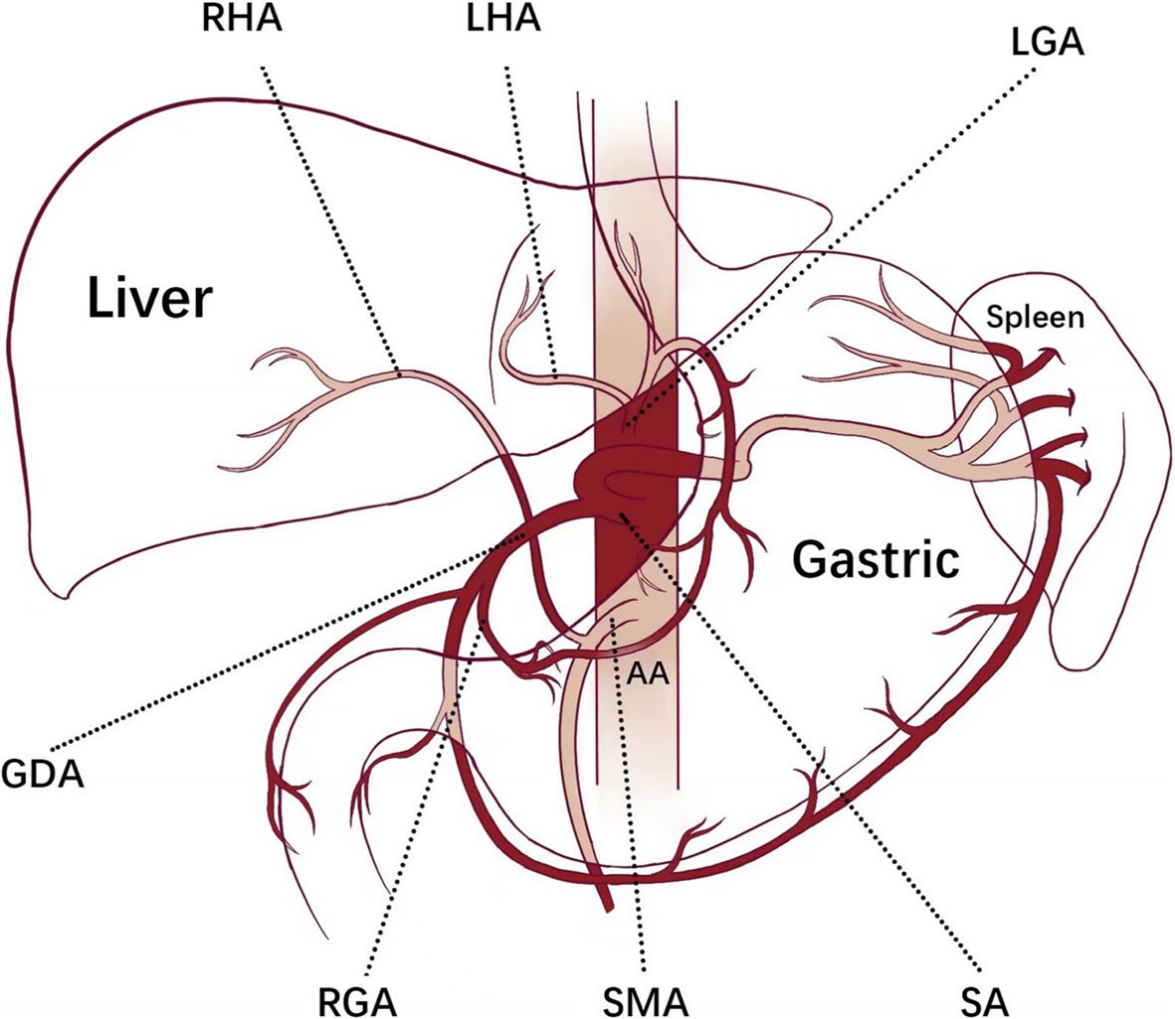
It visually depicts the variation of the blood vessels supplying the liver, stomach, and spleen from the abdominal aorta and the vascular network formed by them in this patient

**Fig. 3:**
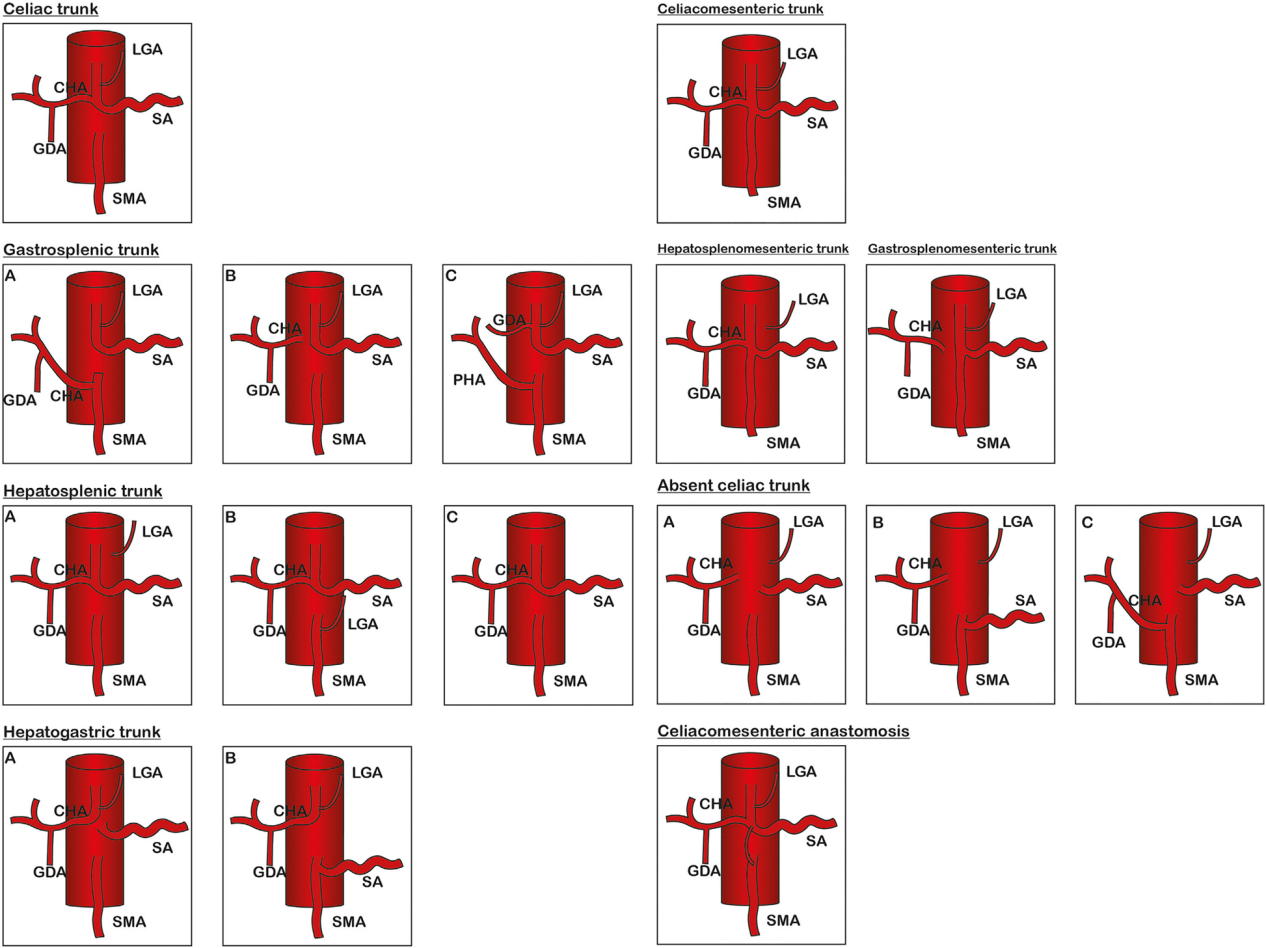
This picture is quoted from Adam Whitley’s study [3] and summarizes the various types of celiac trunk variations from 17391 cases with celiac trunk mutations. Our patient belongs to the category of without celiac trunk, but the mutation of our patient is not mentioned

A 58-year-old woman was admitted to our department for surgical treatment of GC. Endoscopic wight-light images demonstrated a 3–4-cm irregular bulge lesion with an ulcer-like central depression located on the greater curvature of the gastric antrum. Histopathological diagnosis was poorly differentiated carcinoma. IHC:CDX2 (+), CKpan (+), EMA (+), Ki67 (+10%), CgA (−), and Syn (−). Persistent upper abdominal pain was the main symptom. The signs of abdominal physical examination were negative. Results of subsequent laboratory tests were within normal limits. Contrast-enhanced CT first indicated the absence of both CA and CHA. Therefore, we used computerized 3D vascular reconstruction technology to reconstruct the abdominal trunk and its branch vessels and confirmed this extremely rare vascular anomaly before operation. Both CA and CHA were absent. SA and GDA originated from AA. And LGA originated from AA directly above the junction of the SA and GD and gave off LHA. RHA originates from SMA (Figs. 1, 2, and 3)

Complete laparoscopic radical gastrectomy with D2 lymph node dissection and Billroth II digestive tract reconstruction has been performed. During the operation, the absence of both CA and CHA while RHA arising from SMA and LHA arising from the LGA was found. The tumor was located in the lesser curvature of the antrum and invaded the serosa. Prominent fused enlarged lymph nodes were reached in nos. 5 and 6. Postoperatively, the specimen showed that the tumor was located in the gastric antrum, infiltrating ulcerative type and covered with pus filth, with a size of about 4 × 3 cm. Postoperative histopathological diagnosis was ulcer-type (size 3 × 2.9 × 0.7 cm), moderately-poorly differentiated adenocarcinoma, infiltrating into the submucosa and metastasizing to 1/17 lymph nodes on the lesser curvature and 5/7 lymph nodes on the greater curvature. IHC:CDX-2(+), CK7(+), CK20(+), Ki67(+70%), CgA(−), Syn(−), CD56(−), P53(+), P16(−), Villin(+), and Contrast/CerbB-2(−).

The technical parameters of CT were as follows: Imaging was performed using a Canon Aquilion ONE (Toshiba Medical Systems, Tokyo, Japan) which was equipped with 320 detector rows and with each 0.5 mm in width. The scanning range was 2–3 cm from the lower edge of the diaphragm to the upper edge of the pubic symphysis. The scan was helical with a 2.0-mm section thickness (120 kV, 300 mA), gantry speed 0.625 s/rotation and table speed 25.5 mm/rotation. Images were reconstructed in the axial plane with 1 mm slice thickness. The intravenous iodinated contrast was iohexol (370 mg/mL; Omnipaque, GE Healthcare, Shanghai). Seventy milliliters was injected at 5 mL/s. Acquisition was triggered automatically by an attenuation of 100 HU in AA.

## Discussion

The celiac trunk trifurcating into three main branches (LGA, SA, and CHA) is the most classic and common. It was described by Haller and called tripus Halleri in 1756 [1, 2]. In Adam Whitley’s study, the prevalence of complete CA was 89.15%, the prevalence of variant CA anatomy was 10.85%. The pooled prevalence of the incomplete CA, absence, and other types was 8.71%, 0.28%, and 1.86% respectively. In the absence of CA, LGA, CHA, and SA were all derived from the AA respectively. Or one of the three vessels originated from SMA and the other two vessels originated from AA [3]. In the Soon-Young Song study, 96.30% of the cases had normal CHA anatomy, and 3.71% had a variation in the origin and/or anatomical process of the CHA. Among the cases with CHA variants, the absence of CHA account for 1.10%, GDA originated alone without hepatic artery component in the absence of CHA cases, and the most common origin of GDA was SMA [4]. We first reported the case with the absence of both CA and CHA. And LGA originated directly from the AA. SA and GDA originated from AA and joined together. LHA originated from LGA, and RHA originated from SMA. To our knowledge, no such variation has been described in the worldwide literature.

The limitation of this paper is that the abdominal angiography could not be performed before surgical operation, but this vascular anatomy was reconstructed by the computer 3D reconstruction technology. Since the operation was a laparoscopic radical gastrectomy, the surgical field of view also have a few limitations. It is a pity that the images of the mutant blood vessels and their adjacent blood vessels could not be fully exposed.

Accurately understanding the vascular anatomy of the CA is essential for surgery on the stomach, pancreas, spleen, liver and biliary tree. Identifying vascular variation prevents intraoperative confounding and complications [5]. Before any major surgery on the upper gastrointestinal organs, preoperative Multi Detector CT angiography and 3D reconstruction should be performed to identify all vascular variants for optimal preoperative planning. Celiac trunk anatomy is pivotal in the surgical field. And it is equally important for some interventional radiology procedures, such as endovascular treatment of aneurysms, angioplasty and stent placement of mesenteric ischemia, arterial embolization for bleeding control, and chemoembolization for liver tumors [6].

In conclusion, our case demonstrated a novel, undescribed, and unclassified vascular anatomy. We hope provide new tips for the field of surgery and interventional radiology.

## Data Availability

All patient data and clinical images adopted are contained in the medical files of Shaoxing People’s Hospital. The data supporting the conclusions of this article are included within the article and its figures.

## Abbreviations

GC: Gastric cancer
CT: Computed tomography
CA: Celiac trunk artery
CHA: Common hepatic artery
3D: Three-dimensional
SA: Splenic artery
GDA: Gastroduodenal artery
AA: Abdominal aorta
LGA: Left gastric artery
LHA: Left hepatic artery
LGA: Left gastric artery
RHA: Right hepatic artery
SMA: Superior mesenteric artery
